# Investigation into pedestrian exposure to near-vehicle exhaust emissions

**DOI:** 10.1186/1476-069X-8-13

**Published:** 2009-03-30

**Authors:** Neil A Buzzard, Nigel N Clark, Steven E Guffey

**Affiliations:** 1 Department of Mechanical and Aerospace Engineering, West Virginia University, Engineering Sciences Building, Evansdale Drive, Morgantown, WV 26506, USA; 2 Department of Industrial and Management Systems Engineering, West Virginia University, Mineral Resources Building, Evansdale Drive, Morgantown, WV 26505, USA

## Abstract

**Background:**

Inhalation of diesel particulate matter (DPM) is known to have a negative impact on human health. Consequently, there are regulations and standards that limit the maximum concentrations to which persons may be exposed and the maximum concentrations allowed in the ambient air. However, these standards consider steady exposure over large spatial and time scales. Due to the nature of many vehicle exhaust systems, pedestrians in close proximity to a vehicle's tailpipe may experience events where diesel particulate matter concentrations are high enough to cause acute health effects for brief periods of time.

**Methods:**

In order to quantify these exposure events, instruments which measure specific exhaust constituent concentrations were placed near a roadway and connected to the mouth of a mannequin used as a pedestrian surrogate. By measuring concentrations at the mannequin's mouth during drive-by events with a late model diesel truck, a representative estimate of the exhaust constituent concentrations to which a pedestrian may be exposed was obtained. Typical breathing rates were then multiplied by the measured concentrations to determine the mass of pollutant inhaled.

**Results:**

The average concentration of diesel particulate matter measured over the duration of a single drive-by test often exceeded the low concentrations used in human clinical studies which are known to cause acute health effects. It was also observed that higher concentrations of diesel particulate matter were measured at the height of a stroller than were measured at the mouth of a mannequin.

**Conclusion:**

Diesel particulate matter concentrations during drive-by incidents easily reach or exceed the low concentrations that can cause acute health effects for brief periods of time. For the case of a particularly well-tuned late-model year vehicle, the mass of particulate matter inhaled during a drive-by incident is small compared to the mass inhaled daily at ambient conditions. On a per breath basis, however, the mass of particulate matter inhaled is large compared to the mass inhaled at ambient conditions. Finally, it was determined that children, infants, or people breathing at heights similar to that of a passing vehicle's tailpipe may be exposed to higher concentrations of particulate matter than those breathing at higher locations, such as adults standing up.

## Background

There is extensive literature that supports the relationship between atmospheric diesel particulate matter (DPM) and adverse human health effects [1-11]. Consequently, standards have been set to regulate the allowable level of ambient particulate matter and limit the maximum concentration to which persons can be exposed. In the U.S., airborne particulate matter less than 2.5 microns in size (PM_2.5_) is required to be at or below 35 μg/m^3 ^over a 24 hour period and an annual arithmetic mean of 15 μg/m^3 ^[12]. However, those air quality standards address exposures averaged over large spatial (greater than 100 meters) and time (24 hours) scales. In addition, the Mine Safety and Health Administration (MSHA) set an occupational exposure limit on diesel particulate matter of 160 μg/m^3 ^averaged over an 8 hour period, effective May 2008 [13]. Because it is estimated that 35% of ambient PM_2.5 _typically is contributed by mobile sources [14], there has been interest in the possibility of health effects due to elevated exposures near roadways [15]. In literature, the term "near roadway" refers to the distance from a roadway at which pollutants are measured, typically less than 300 meters. This paper is concerned with exposures that are even closer to the vehicle exhaust than the distances termed "near roadway" in the literature. In fact, this paper uses the term "near vehicle" to show the closeness of the exposure to the passing vehicle.

While most automobile exhausts are directed to the rear of the vehicle, many vehicles around the world, specifically pickup trucks, employ tailpipes that direct exhaust towards the passenger side of the vehicle. Since right side tailpipes direct emissions towards U.S. sidewalks and roadsides, there is reason to be concerned that people on sidewalks and near roadways are exposed to hazardous exhaust constituents at levels greater than typical "near roadway" levels. Because their faces are closer to the level of tailpipes, children and babies in strollers could be even more heavily exposed.

When exhaust is emitted from a diesel vehicle, it can be characterized as a plume of particles and gaseous materials. It is plausible that within such plumes particle concentrations may substantially exceed regulations, especially for brief periods. Wind tunnel studies [16,17] show that there may not be any appreciable evolution of particle sizes within a plume and that dilution ratios can range from 75 to 125 at a distance of 8.5 meters downstream of the tailpipe. In contrast to these findings, a vehicle chase study [18] observed actual dilution ratios as large as 1,000:1 in two seconds. However, the chase study was conducted at freeway speeds of 40 to 55 mph, which is more than double the local street speeds (20 to 25 mph) tested in this study and did not consider the stop and go traffic often encountered on busy streets. Therefore, it is reasonable to believe that dilution rates would be far lower at local street speeds for two reasons: (i) the travel time for the plume to reach a sidewalk is very short, providing little time for appreciable dilution, and (ii) the turbulence imparted to surrounding air by slow-moving vehicles would be relatively low. Hence, it is plausible that pedestrians may be exposed to diesel particulate matter concentrations that are high enough to cause acute health effects, though perhaps for brief periods as each vehicle passes a pedestrian or idles at the sidewalk. In addition, since pedestrians are generally walking in front of stores, shops, and other buildings, it is quite likely that concentrations would build over time, especially when traffic is heavy or stop and start.

Although there is a limited amount of information regarding acute and short-term (e.g. less than 8 hours) exposures to diesel exhaust, there is strong evidence from human and animal studies that exposures to low concentrations of diesel exhaust (300 μg/m^3^) can cause pathophysiological symptoms such as particle accumulation in the lungs, acute eye, nasal and throat irritation, neurophysiological symptoms such as lightheadedness and nausea, and respiratory symptoms including cough and phlegm [11]. Since data from dilution tunnel measurements [19-21] can not accurately simulate this type of actual human exposure, drive-by experiments are necessary.

The vehicle studied here was a diesel-fueled pickup truck, which has an original tailpipe that discharges directly toward the sidewalk. Multiple scenarios, including exposure to a simulated adult or a child in a stroller beside the road, were examined for different vehicle operating conditions during the "drive-by." The concentrations of diesel particulate matter that reach a pedestrian's mouth were measured and compared to both ambient pollution levels and to concentrations used in human clinical exposure studies. In addition, both the short-term, time-weighted average exposure and the cumulative mass of particulate matter potentially inhaled per event were quantified.

## Methods

### Study site

The study was conducted during August 2007 in Morgantown, West Virginia on a two-lane road bisecting the WVU Evansdale campus. The study location was chosen for its minimal traffic congestion and the presence of an adequate power supply within 31 m. Additionally, the land on one side of the road was relatively flat for about 5 m, providing a simple topography that should be comparable to many urban or suburban sites. There was a single building nearby that could have caused a small urban canyon effect; however, preliminary measurements revealed that background concentrations of pollutants of interest were very low. This led to the belief that the building would not prevent dispersion of the vehicle exhaust or affect measurements.

### Apparatus

Particulate matter (PM), carbon monoxide (CO), carbon dioxide (CO_2_), and oxides of nitrogen (NOx) were measured continuously using a Cambustion DMS500 Fast Particle Spectrometer, a Horiba AIA-220 CO/CO_2 _analyzer, and an EcoPhysics CLD-822 NOx analyzer, respectively. The Cambustion DMS500 Fast Particle Spectrometer is a mobility-based particle sizing instrument used to measure or count particles between 5 and 1,000 nanometers in mobility diameter. The DMS500 operates by charging each particle precisely using a corona discharge as it flows into a strong electrical field contained inside a classifier column. This electrical field then deflects the particles towards the electrometer detectors depending upon each particle's aerodynamic drag/charge ratio (mobility). When the particles contact the detectors at different points throughout the column, the increase in current due to each particle's charge is measured. The outputs from the 22 electrometers are then processed in real time to provide spectral equivalent diameter data and other desired particle parameters.

Although the particles in diesel exhaust do not have a constant density and are not always spherical, both spherical shape and constant density are typically assumed when estimating mass using sample data from instruments designed to measure particle number-weighted size distribution via particle mobility [22]. For example, the TSI Engine Exhaust Particle Sizer™, a mobility-based instrument that operates on the same principle as the DMS500, requires the assumption of spherical particles of unit density to calculate mass [23]. Mobility-based instruments are based on Stokes' law which can be used to determine each particle's equivalent spherical diameter which is equivalent to the diameter of a spherical particle of unit density with the same settling velocity as the collected particles [22].

Though this approach has been used by some [23], others have instead developed more accurate correlations between particle size and mass. A recent study [24], which compared the DMS500 and a Scanning Mobility Particle Sizer (SMPS), showed the development of the following correlation between particle size and mass for the DMS500:(1)

where D_eme _is the electrical mobility equivalent diameter in nanometers. Both of these approaches for calculating mass from DMS500 particle number data were applied to the data from this study and compared to each other.

The Horiba AIA-220 analyzer, installed in a custom measurement system, measured CO and CO_2 _continuously using nondispersive infrared (NDIR) technology. The custom measurement system, housed inside a stainless steel container, consisted of a pump to control and provide adequate sample flow, a particulate filter to remove any particles that may damage the instrument, and a cooler to remove all water vapor from the sample. Including the analyzer, the measurement system used a sample flow rate of about 7 liters per minute. The EcoPhysics CLD-822 analyzer measured NOx continuously using the principle of chemiluminescence. This analyzer was used separately, without a custom measurement system and used a flow rate of approximately 2 liters per minute.

Although the Horiba and EcoPhysics analyzers are often considered to be continuously integrated and nearly instantaneous, they are not. In a typical emissions measurement laboratory setup, these analyzers have been found to have response delays up to 15 seconds [25]. However, the bulk of these delays are not caused by the analyzers alone, rather, they are also due to the flow of exhaust through a vehicle's exhaust system and travel time through the emission sampling system. According to manufacturer user manuals, the Horiba and EcoPhysics analyzers have delays of 0.5 to 10 s and less than 1 s, respectively, from the time a sample enters the analyzer until it is detected by the sensor. The sample tubing used in this study was approximately 5 m long with an inner diameter of 0.32 cm. The transport delays for the analyzer sampling systems were calculated using this information and Equation 2:(2)

where T_D _is the transport delay, V is the volume of the sample line and sampling system, and F is the sampling system flow rate. The transport delays were calculated to be approximately 3 and 11 seconds for the Horiba and EcoPhysics sampling systems, respectively. The time delays between sample collection at the probe inlet and sensor response are given by the manufacturer specifications stated previously and are in addition to the transport delays presently discussed.

Before testing began, the gaseous analyzers were calibrated using gases of known concentration and linear regression using 11 points over a range from 0 to 2.003% for the CO_2 _analyzer, 0 to 99.8 ppm for the CO analyzer, and 0 to 101 ppm for the NOx analyzer. To do this, both the known concentrations and their corresponding analog voltage outputs were recorded. Quadratic polynomial regression equations were developed from the calibrations and used to predict concentrations from measured voltages.

The vehicle in the test was a 2006 Dodge Ram 2500 with a 5.9 L, 325-hp diesel engine that discharges its combustion products through an exhaust system outfitted with an oxidation catalytic converter. The tailpipe discharges towards the passenger side of the vehicle behind the rear wheel. The vehicle had Shell Rotella 15W40 oil and used ultra-low sulfur diesel pump fuel. An AutoTap OBDII Diagnostic Scanner connected to an on-board laptop computer running AutoTap software was used to monitor and record broadcast engine control unit (ECU) data. The software allowed the vehicle operator to monitor in real-time the calculated percentage load which made it possible to maintain the desired engine loads of 100%, 50%, and 0% when passing the sampling location. These loads were chosen to simulate real world driving conditions such as hard acceleration, medium acceleration, and deceleration.

**Figure 1:**
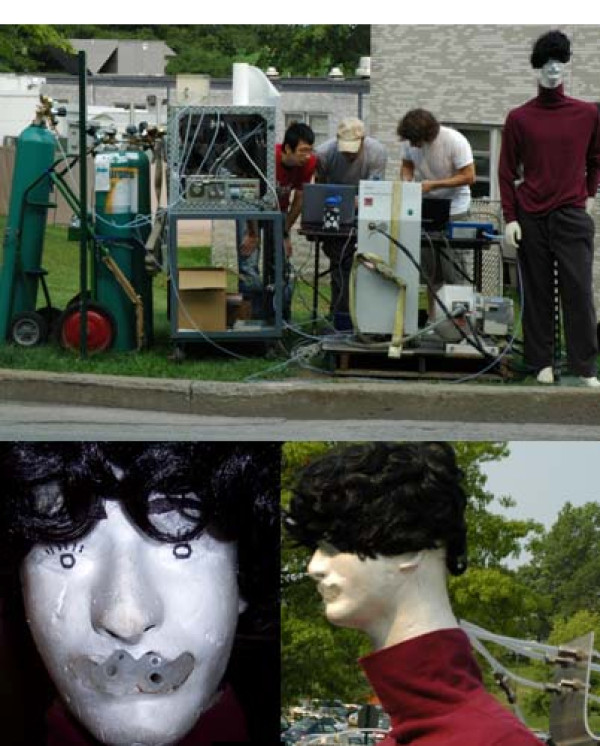
**Mannequin and test setup**. The top photograph shows the emissions measurement equipment and the mannequin alongside the test road. The bottom photographs show the mannequin's head with sample tubes in the mouth and their attachment to the transport tubing.

At the sampling location, a life-like mannequin with a detachable Styrofoam™ head was used as a surrogate for human pedestrians. The mannequin along with the emissions measurement equipment can be seen in Figure 1. Including the head, the mannequin was about 1.8 m tall (i.e. approximately 1.65 m at the mouth). Sample probes, located in the mouth of the mannequin, extended though the back of the Styrofoam™ head and attached to Teflon transport tubing 5 m in length connected to the analyzers. To mimic a child in a stroller, the mannequin's head, sample probes and all, were placed inside a stroller and positioned near the roadway. During this setup, the sample probes were approximately 0.85 m from the ground.

Because particulate matter formation and movement are greatly affected by meteorological conditions, the temperature, pressure, wind direction, and relative humidity were recorded prior to each test run. The ambient temperature and relative humidity measurements were obtained from a handheld indoor/outdoor thermometer from RadioShack. Barometric pressure measurements were taken using a Heise PTE-1 handheld digital calibrator with a module having a calibration range of 0–30 psi. Wind direction was monitored by recording the direction in which a streamer pointed before each test run, using a handheld compass. Wind speed data were not obtained.

### Scenarios observed

Although the term "pedestrian" typically refers to a person walking or traveling on foot, for the purposes of this study the term was broadened to include persons who are near roadways and either walking or standing. Also, the term does not distinguish between individuals who are nearby for occupational or personal reasons. Analyzers were placed along the test road to measure exhaust constituents at the mouth of a surrogate pedestrian. During periods of sampling, a diesel fueled vehicle was driven past the mannequin and the analyzers under different operating conditions.

Though there have been several studies to analyze near-tailpipe vehicle exhaust or near-roadway exposure [26-29], few have instrumented the vehicle to record ECU data during the drive-by incidents, as was done in this study. The mannequin, which has been fully described above, was placed beside the roadway to simulate an adult male standing on the sidewalk next to a roadway. In addition, the mannequin's head was removed and placed in a stroller to simulate a child in a stroller.

The three operating conditions varied when the test vehicle passed the sampling location were (i) acceleration at 100% (full) load, (ii) acceleration at 50% (part) load, and (iii) cruising at a constant velocity with high engine speed. These operating conditions were chosen because all three commonly occur near pedestrians and could be expected to produce very different levels of diesel particulate matter expulsion. Several studies [30-32] have shown that the formation of particulate matter and other diesel exhaust constituents varies greatly with engine operation. The highest particulate concentrations and emission rates observed in the third study were linked to heavy engine acceleration, high engine speed, and high torque [32].

The acceleration tests were accomplished by accelerating the vehicle from a rolling start (5 mph) past the sampling location while monitoring the engine load to ensure that the proper load was maintained while the vehicle passed the sampling location. The cruising tests were accomplished by accelerating the vehicle to approximately 25 mph and maintaining a constant speed for at least 30 meters before passing the sampling location. During these drive-by tests, the vehicle operator attempted, when near the sample locations, to keep the vehicle at a distance of about 0.5 m from the curb and thus about 0.75 m horizontally from the sample probes. Four to six drive-bys were conducted for each combination of scenarios.

### Pollution monitoring

Teflon sample lines from the analyzers were connected directly to the mannequin's mouth and run roughly 5 m to sampling devices. Air was sampled continuously providing continuous emissions data as the test vehicle passed the mannequin during each test run. Data from all of the analyzers were recorded simultaneously by connecting the analog outputs of the Horiba and EcoPhysics analyzers to the analog inputs on the Cambustion DMS500 via modified coaxial cable. A program, supplied with the Cambustion DMS500, was used both to control the DMS500 instrument and to record the PM, CO, CO_2_, and NOx measurements. The resulting measurements were associated with specific test vehicle engine conditions by synchronizing the computer used to record analyzer measurements with the computer recording engine data. The time at which the vehicle passed the sampling point was recorded using a Microsoft Visual Basic™ program custom written for this study. Using these time-stamped data, the engine conditions during each test could be correlated with the emissions measured during each test. To ensure that emissions linked with the test vehicle were not affected by other vehicles, the drive-by runs were conducted when there were no other vehicles nearby.

Because the particle size range of the DMS500 typically accounts for 80 to 95 percent of the total particulate matter mass found in diesel exhaust [33], the resulting measurements can be assumed to approximate PM_2.5 _concentrations. Thus, PM measurements were taken to estimate PM_2.5 _exposure, CO was measured as an attempt to correlate CO concentrations with PM concentrations (see [34]), and CO_2 _and NOx were measured to in an attempt to quantify the dilution ratio of the exhaust exiting the test vehicle's tailpipe.

### Data reduction

Once all the data had been acquired from testing, a program custom-written in Microsoft Visual Basic for Applications (VBA)™ was used to extract the desired data from text files and import it into a Microsoft Excel™ file. During extraction, the gaseous sample concentrations where calculated from the observed voltages using the corresponding calibration equations. In addition, the concentration of particulate matter in micrograms per cubic meter of air (μg/m3) was computed using the previously mention analyses correlating particle diameter and mass.

A vehicle's exhaust system and the emission sampling system have considerable time delays (up to 12 seconds combined) [25,35,36], although raw exhaust measurements, such as obtained in this study, typically have shorter time delays than standard dilution tunnel measurements [36]. Consequently, all gaseous data had to be corrected based on the analyzer specifications as well as the measured sampling delays. By combining the sample tube transport delays and manufacturer specified delays, the delay for the CO and CO_2 _data was estimated to be approximately 3.5 seconds, and the delay for the NOx data was estimated to be about 12 seconds. These delays were required in order to correlate the emissions with specific engine operating conditions; however, the instantaneous pollutant concentration data obtained from analyzers are diffused in time [36] because they do not represent the instantaneous emissions that may arise due to a short lived engine operating condition. No corrective measures were taken to rectify the data because the nature of the diffusion was unknown.

### Exposure estimation

Human exposure to diesel exhaust is typically considered as an average particulate matter concentration over a certain amount of time. For example, the MSHA exposure limit is a concentration averaged over an 8 hour period [13]. During drive-by incidents, the measured concentration of particulate matter is initially equivalent to the background concentration but quickly increases to a maximum as the exhaust plume reaches the sample lines. It then decreases back to the background concentration levels as the exhaust is diluted by mixing with ambient air.

In order to estimate the exposure pedestrians may experience, the instantaneous sample concentrations of diesel particulate matter obtained at 5 Hz from the DMS500 were mathematically averaged over the duration of each drive by incident. For this study, the duration of an incident was defined as the time interval beginning when the exhaust plume from the tailpipe produced a noticeable increase in particulate matter concentration at the computer and ending when the exhaust plume had diluted sufficiently that the measured concentration of particulate matter was near background levels again. The noticeable increase in concentration or beginning of an event was determined by first computing the standard deviation of the background concentration for two to four seconds starting at the events time stamp. A three point running average of the concentration was then computed. If this value was greater than the average background concentration plus 10 times the standard deviation of the background concentration, the time associated with the second point in the three point average was considered to be the time the event began. The end of the event was similarly determined to be when the value of the running average was less than the average background concentration plus 10 times the standard deviation of the background concentration.

To compare the estimated mass inhaled during the drive-by incidents with the estimated mass inhaled at ambient conditions, maximum and minimum ambient conditions were specified. The ambient concentrations considered were equivalent to (i) the National Ambient Air Quality Standard and (ii) the ambient concentration in Darrington, Washington, a city which the EPA considers to have good air quality. These ambient concentrations of 35 and 5 μg/m^3^, respectively, represent reasonable maximum and minimum expected ambient concentrations of particulate matter.

Since the number of drive-bys in the study was fewer than the number pedestrians may experience on city sidewalks, the estimated exposures for typical pedestrians were determined by multiplying the average test values by a reasonable estimate of typical frequency of drive-bys. For the latter, it should be noted that the theoretical maximum traffic volume a single lane road can support is given by the ratio of vehicle speed and vehicle spacing [37]. Assuming a speed limit of 25 mph, such as that of the test road, and a vehicle spacing of 12.2 meters (approximately two car lengths), the maximum traffic volume that can be obtained is 3,300 vehicles per hour. Of course, not all vehicles are diesel-powered and gasoline engines also emit particles at measurable mass and number levels [38,39]; particle mass and number emissions from gasoline vehicles are orders of magnitude smaller than from diesel vehicles. Using the assumptions made in the EPA's MOBILE6 emissions model, it was determined that 1.02% of all light duty vehicles and class 2B and class 3 heavy duty vehicles produced in the U.S. in 2008 were diesel fueled [40]. This percentage implies that on average 33 diesel fueled vehicles pass a single point on a road, such as that described above, every hour. Therefore, over an 8 hour period, a typical incident count could be as high as 264. Note that this 8 hour time period comes from an occupational exposure standard.

Another type of short-term exposure considered to be applicable to drive-by incidents, such as explored in this study, is exposure per inhalation. This type of short-term exposure deals with single inhalations of very high concentrations. To determine this exposure as a worst case scenario, an incident from each scenario with the highest instantaneous concentrations was aligned with the inspiration of a breath. Inspiration lengths of 2.5 and 1.5 seconds were used to imitate walking and standing breathing rates, respectively. This maximum amount of particulate matter inhaled in a single breath during a drive-by incident was then compared to the amount of particulate matter inhaled in a single breath at the same ambient conditions stated previously.

## Results

### Drive-by test results

**Figure 2:**
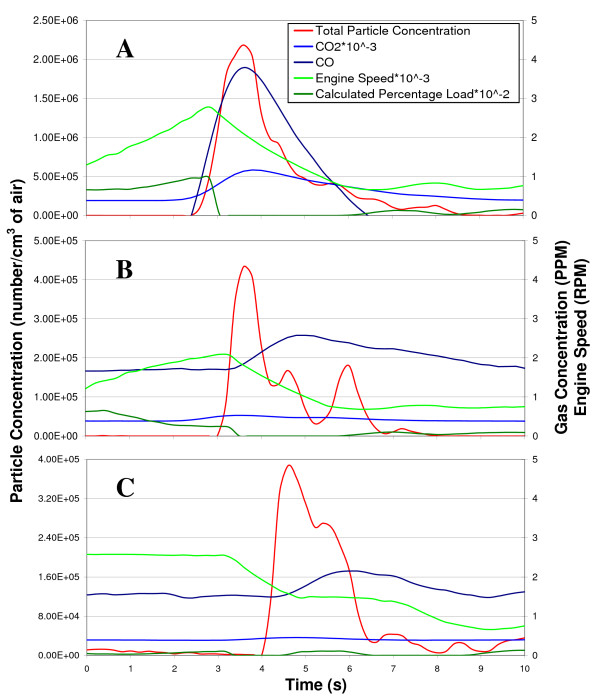
**Raw drive-by test data**. The raw data from drive-by tests in which the test vehicle was driven past a mannequin are plotted versus time. Plot A represents the case involving accelerating the test vehicle past the sample location at full load. Plot B corresponds to the case where the test vehicle accelerated past the sample location at part load. Plot C shows the case in which the test vehicle was driven past the sample location at a constant speed with high engine speed and almost zero load.

The raw data from typical drive-by tests can be seen in Figure 2. Figure 2A represents the case where the test vehicle accelerated past the mannequin at full load. Figure 2B corresponds to the case where the test vehicle accelerated past the mannequin at part load. Figure 2C shows the case in which the test vehicle was driven past the mannequin at a constant velocity with high engine speed and low load. In these figures, the driving conditions of the test vehicle, namely engine speed and calculated percentage load, as well as the concentrations of the desired exhaust constituents at the mouth of the mannequin were plotted versus time.

The average incident particulate matter concentrations observed and the corresponding incident durations for the 100% load acceleration tests can be seen in Table 1. The average particulate concentrations measured throughout the full load acceleration tests were 124.97 and 199.16 μg/m^3 ^for the adult pedestrian and child in stroller scenarios, respectively. The average duration of these incidents was determined to be approximately 5.1 and 12.67 seconds, respectively. The maximum concentrations averaged over 1.5 seconds to simulate a poorly timed breath observed for the adult pedestrian and child in stroller scenarios were 613 and 1,114 μg/m^3^, respectively. The maximum concentrations averaged over 2.5 seconds for a similar breath observed for the adult pedestrian and child in stroller scenarios were 396 and 860 μg/m^3^, respectively.

**Table 1:** Average incident concentrations and corresponding durations

Scenario	Run	Average Incident Concentration (μg/m^3^)	Incident Duration (s)
Mannequin	1	87.50	5.4
	2	84.99	6.6
	3	104.82	4.4
	4	227.92	5.8

Stroller	1	364.27	8.6
	2	233.88	6.4
	3	238.72	9.2
	4	245.70	10.8
	5	200.45	15.4
	6	139.86	6.8

### Exposure

Breathing rates for men, women, and children both walking slowly and standing still, (see Table 2), were obtained from a study [41] produced by the California Air Resources Board and multiplied by the average incident concentrations. In this way, the mass of diesel particulate matter inhaled per drive-by incident by men, women, and children could be computed. To see if this inhaled amount was significant relative to the amount normally inhaled over a 24 hour period, the daily mass of particulate matter inhaled by pedestrians at different ambient concentrations was calculated. These values can be seen in Table 3.

**Table 2:** Breathing rates for adults and children in LPM

Activity	Adult Male	Adult Female	Children
Walking	24	20	14
Standing	11	8	7

**Table 3:** Mass inhaled in μg from 264 drive-by incidents and daily inhalation at ambient levels.

Scenario	Drive-by		NAAQS		Darrington, WA	
**Mannequin**	**Adult Male**	**Adult Female**	**Adult Male**	**Adult Female**	**Adult Male**	**Adult Female**

Walking	72.58	60.49	1209.60	1008.00	172.80	144.00
Standing	33.27	24.19	554.40	403.20	79.20	57.60

**Stroller**	**Child**		**Child**		**Child**	

Walking	116.55		705.60		100.80	
Standing	58.27		352.80		50.40	

**Figure 3:**
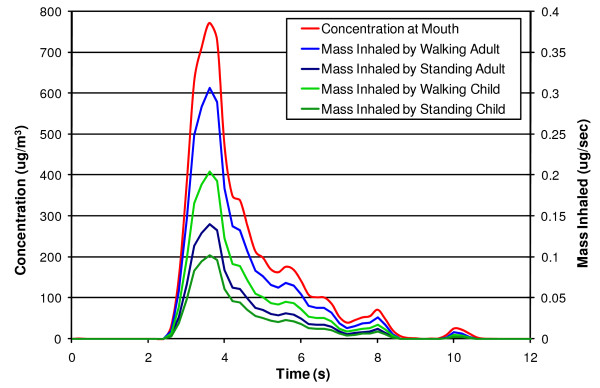
**PM concentration and mass inhaled by a pedestrian for a typical acceleration test**. Once the data was acquired following a typical drive-by acceleration test, particle size-mass correlations and human breathing rates were applied to the data. The red line in the figure represents the PM concentration measured at the mannequin's mouth. The light blue line corresponds to the mass inhaled using the breathing rate of an adult walking. The dark blue line represents the mass inhaled using a breathing rate of an adult standing still.

The measured concentration of particulate matter and the corresponding mass inhaled by an adult male pedestrian and a child when walking and standing (or sitting) during a typical drive-by incident can be seen in Figure 3. The amount of diesel particulate matter inhaled by adults and children after being subjected to 264 average drive-by incidents was calculated and can be seen in Table 3, which also shows the PM mass inhaled daily at ambient levels. In addition to effects of multiple average drive-by incidents on daily inhalation, the effects of per inhalation exposure were considered. For this analysis, incidents from each scenario with the highest particulate concentrations observed were aligned with the inspiration of a breath. To determine if this inhaled mass was significant, it was compared to the calculated mass of PM inhaled per breath at the same ambient conditions mentioned previously. These values and the mass inhaled in a single breath during the worst drive-by test observed are shown in Table 4.

**Table 4:** Maximum mass in μg inhaled per breath during worst drive-by and at ambient levels.

Scenario	Drive-by		NAAQS		Darrington, WA	
**Mannequin**	**Adult Male**	**Adult Female**	**Adult Male**	**Adult Female**	**Adult Male**	**Adult Female**

Walking	0.1583	0.1317	0.0350	0.0291	0.0050	0.0042
Standing	0.1123	0.0818	0.0096	0.0070	0.0014	0.0010

**Stroller**	**Child**		**Child**		**Child**	

Walking	0.2293		0.0233		0.0033	
Standing	0.1300		0.0061		0.0009	

## Discussion

Although Figure 2 only shows one typical plot obtained from each driving condition considered, it can be seen that the curves representing instantaneous particulate matter concentration are quite different in each plot. This is because peak concentrations and durations of exposure incidents varied widely from test to test and because truck operation differed from case to case. Wind speed and direction greatly affected the peak concentrations measured and the durations of the exposure incidents by affecting the exhaust travel time from the tailpipe to the sample lines and varying the exhaust dilution and amount of residual entrainment in eddies. The vehicle operator also affected the repeatability of tests due to varied pedal commands and vehicle positioning with respect to the sampling point.

As discussed previously, two methods for computing the particulate mass were utilized in this study. One method was specifically developed for the DMS500 while the other is typical of mobility based particle sizing instruments. When comparing the results using these two methods, it was found that the mobility based method was consistently 1.4 times higher than the DMS specific method. For particles below 1 μm, the assumption that the particles have the same velocity as the air stream becomes invalid [22]. Consequently, the particles can slip between air molecules requiring the application of a Cunningham correction factor [22]. By applying a Cunningham correction factor of 0.7 for rough spheres [22], good correlation between the two methods for calculating particle mass was obtained. Though this correction factor allowed for good correlation between the two methods, the DMS specific method for calculating particulate mass was used throughout the study for computational ease.

From the results in Table 1, it is apparent that particulate concentrations are higher at the height of a stroller. The results from Table 1 also imply that on average a pedestrian would be exposed to average diesel particulate matter concentrations during a drive-by that are near levels used in human clinical studies (e.g. 300 μg/m^3^). Low particulate concentrations such as these have been documented to cause acute health effects including accumulation of particulate matter in the lungs; however, accumulation occurs over longer periods of time. It must be considered, though, that these results were obtained via a simplistic model that represents an in-use minimum vehicle PM expulsion. In reality, a large number of drive-bys would have a more significant effect on exposure because ambient levels of pollutants would build as not all of the pollutants are carried away or dispersed.

While the particulate matter concentrations averaged over incident durations are lower than the concentrations used in many human clinical studies, the maximum concentrations observed for 1.5 and 2.5 seconds (i.e. the length of a breath) were 2 to 3 times higher than the concentrations used in many human clinical studies. This result implies that particulate concentrations from drive-by incidents can easily reach levels that cause acute health affects; however, the duration is still very small compared to the 1 or 2 hour exposures used in clinical studies. Although numerous diesel particulate exposure studies have been conducted on humans [1-11], due to the difficulties faced in assessing the exposure and the corresponding health effects, there has not been enough information to develop standards that address such short exposures. Furthermore, there is not enough information regarding human dose studies to elicit a threshold concentration, beyond which health effects are certain to occur. More research is necessary in each of these areas.

Although the results from this study show that curbside particulate concentrations can easily surpass those used in clinical studies, the fact that the test vehicle is a new model vehicle with a catalytic converter must be taken into consideration. In-use fleet particulate matter emissions vary from vehicle to vehicle. For example, in the E-55/59 California truck emissions inventory program, medium duty trucks were exercised through a transient test cycle, termed MHDTLO, and it was found that a 1990 model year truck emitted particulate matter at a level that was 10.2 times higher than one model year 2000 truck, and 15.3 times higher than another model year 2000 truck [42]. A 1995 truck yielded particulate ratios of 4.5 and 6.3 relative to the two model year 2000 trucks [42]. The 2006 truck used in the present study had low mileage, and it is assumed that the particle emissions represent an in-use minimum for vehicles prior to 2007 models with exhaust filtration. It is reasonable to believe that many in-use trucks would yield particulate levels substantially higher than those yielded by the 2006 diesel pickup.

From Figure 3, it can be ascertained that adults inhale more particulate mass per second then children; however, it must be noted that children have smaller and less developed lungs. It can be seen from Table 3 and Table 4 that in a relatively dirty city, where the ambient pollution level is equivalent to the National Ambient Air Quality Standard, 264 average drive-by incidents can increase the mass inhaled daily by an adult and a child by as much as 6% and 17%, respectively. At the same ambient particulate concentrations an adult and a child could inhale as much as 12 and 21 times more diesel particulate matter mass, respectively, in a single inhalation during a drive-by incident than at ambient conditions. In a clean city, however, the same exposure can increase mass inhaled by as much as 42% and 116% and as much as 82 and 149 times more mass per breath than at ambient conditions. However, without short-term health effects understanding, it is not possible to project the effect of brief, highly elevated particulate matter levels on a roadside pedestrian.

## Conclusion

Diesel particulate matter concentrations during drive-by incidents easily reach or exceed the low concentrations that can cause acute health effects for brief periods of time. For the case of a particularly well-tuned late-model year vehicle, it was determined that the observed mass of particulate matter inhaled during a drive-by incident was small compared to the mass inhaled daily at ambient conditions. However, on a per breath basis the mass of particulate matter inhaled is large compared to the mass inhaled at ambient conditions. Exposure to actual in-use vehicles could be orders of magnitude greater than the results obtained in this study. It was also determined that children, infants, or people breathing at heights similar to that of a passing vehicle's tailpipe may be exposed to higher concentrations of particulate matter than those breathing at higher locations, such as adults standing up. Based on these results it appears that exposure is likely related to position relative to a vehicle's tailpipe as well as meteorological conditions.

## List of abbreviations

Cm: centimeter; CO_2_: carbon dioxide; CO: carbon monoxide; D_eme_: electrical mobility equivalent diameter; DPM: Diesel Particulate Matter; ECU: engine control unit; EPA: Environmental Protection Agency; F: flow rate; Hz: hertz; LPM: liters per minute; m: meters; MSHA: Mine Safety and Health Administration; NDIR: nondispersive infrared; NOx: oxides of nitrogen; PM: particulate matter; PM_2.5_: particulate matter less than 2.5 microns in size; s: seconds; SMPS: scanning mobility particle sizer; T_D_: transport delay; V: volume; VBA: Visual Basic for Applications; WVU: West Virginia University.

## Competing interests

The authors declare that they have no competing interests.

## Authors' contributions

All three authors were responsible for planning the study and interpreting data. The bulk of the data acquisition and processing was conducted by NAB. NNC provided expertise in the vehicle emissions area, while SEG provided expertise in understanding human exposure to pollutants.
